# Induction of nitric oxide via humming does not improve short-term cognitive performance or influence emotional processing

**DOI:** 10.1371/journal.pone.0301268

**Published:** 2024-04-04

**Authors:** Gregory Francis, Predrag Petrovic, Johan N. Lundström, Evelina Thunell

**Affiliations:** 1 Department of Psychological Sciences, Purdue University, West Lafayette, Indiana, United States of America; 2 Department of Clinical Neuroscience, Karolinska Institutet, Stockholm, Sweden; 3 Monell Chemical Senses Center, Philadelphia, PA, United States of America; Sunway University, MALAYSIA

## Abstract

Nitric oxide (NO) is involved in a variety of biological functions including blood vessel dilation and neurotransmitter release. In animals, NO has been demonstrated to affect multiple behavioral outcomes, such as memory performance and arousal, whereas this link is less explored in humans. NO is created in the paranasal sinuses and studies show that humming releases paranasal NO to the nasal tract and that NO can then cross the blood brain barrier. Akin to animal models, we hypothesized that this NO may traverse into the brain and positively affect information processing. In contrast to our hypothesis, an articulatory suppression memory paradigm and a speeded detection task found deleterious effects of humming while performing the task. Likewise, we found no effect of humming on emotional processing of photos. In a fourth experiment, participants hummed before each trial in a speeded detection task, but we again found no effect on response time. In conclusion, either nasal NO does not travel to the brain, or NO in the brain does not have the expected impact on cognitive performance and emotional processing in humans. It remains possible that NO influences other cognitive processes not tested for here.

## 1 Introduction

Nitric oxide (NO) is a colorless and odorless gas that contributes to a variety of biological functions. In addition to increasing blood flow by signaling blood vessels to relax [1]. NO modulates release of some neurotransmitters [2] (for a review see [3]) and has been connected to a variety of behavioral phenomena in animals [4–7]. One example study [8] investigated the role of NO in rat memory formation for a radial arm maze. One group of rats received a drug (L-NAME) that inhibits synthesis of NO, while another group received a drug (L-arginine) that is a precursor of NO. A third (control) group of rats received only saline or oil injections. Rats receiving L-NAME made more errors (across 30 trials) than the control group, while rats receiving L-arginine made fewer errors than the control group (for the last 10 trials).

In humans, NO is produced in the paranasal sinuses [9], among other places. Considering that NO can cross the blood brain barrier and that humming increases the concentration of nasal NO by releasing it from the paranasal sinuses [10], with a 15-fold increase compared to quiet exhalation, it seems plausible that the released NO might influence the human brain by traveling through the cribriform plate, as is known to occur for other nasally-induced drugs such as oxytocin and insulin [11,12]. Perhaps consistent with such an influence, “active meditation” (which included humming) increased positive affect and decreased negative affect [13]. Various pharmacological promoters and inhibitors of NO have also been explored for possible impacts on emotional processing, especially with regard to anxiety. Recent reviews of the literature on animal models [14,15] reveal a mixed set of findings, suggesting that modifications to NO levels in the brain can, through different mechanisms, either increase or decrease anxiety-related behaviors. All of the studies suggest that NO may act on fear processing circuits, including the amygdala.

Combining the animal and human research, we hypothesized that humming might improve human cognitive performance and impact emotional experience. There are anecdotal reports of humming improving several aspects of mental health (e.g., as part of relaxation techniques such as Bhramari pranayama), possibly through NO production. However, these effects tend to involve long-term impacts of NO, such as improving general health by reducing blood pressure or inhibiting the growth of bacteria and viruses [16]. Here, we test the hypothesis that humming affects short-term cognitive performance by means of NO production using two classic experiments for measuring memory and action/perception. Experiment 1 is based on the classic effect of articulatory suppression [17], where speaking out loud interferes with short-term memory. We anticipated that humming would not have the same detrimental effect on short-term memory as speaking out loud but would instead, given the effects of NO in animal studies, enhance memory performance. Experiment 2 is a speeded response time experiment, which theory suggests should not be directly impeded by humming. Here, we explored whether a faciliatory effect of NO on cognitive processing would produce faster responses. In Experiment 3 we tested the hypothesis that NO levels would impact emotional processing by evaluating possible impacts of humming on emotional evaluations of photos of neutral, positive, negative, or fearful scenes. Finally, in Experiment 4, to avoid task interference effects, we had participants hum just before a speeded response time trial. Experiment 4 also restricted the analyses to participants without congestion and who confirmed they consistently hummed as requested.

For each study, a version of the experiment that runs locally in a web browser and the resulting data is available at the Open Science Framework (https://osf.io/682rv/). All experiments were approved by an internal review board at Purdue University (IRB # 2022–1) and consent was provided online by the participant clicking a button to indicate consent.

## 2 Experiment 1

Our first study is based on the classic effect of articulatory suppression [17] for short-term memory. After viewing a sequence of seven letters, the participant is asked to click on labeled buttons in the same order as the presented letter sequence. On articulatory suppression trials, the participant is asked to say out loud a phrase such as “1 2 3 4 1 2 3 4…” during the letter sequence. Participants are commonly less accurate when reporting the letter sequence on articulatory suppression trials as compared to trials where participants instead quietly observe the letter sequence. According to theories of working memory [18], articulatory suppression occurs because information about the letters is stored in a “phonological loop” that represents speech sounds. Repeating the phrase out loud also uses resources of the phonological loop and so prevents maintenance of letter information. We added a third, humming, condition to an online version of this experiment. Based on the working memory theory, humming should not interfere with the letter information in the phonological loop, so if humming produces an NO-induced advantage, recall of letters should be better as compared to the quiet condition.

### 2.1 Power analysis

To identify an appropriate sample size, we considered previous studies of the articulatory suppression effect. The mean proportion of correctly reported letters is around 0.66 for the quiet condition and around 0.54 for the articulatory suppression condition (online experiment of the articulatory suppression effect [19]) with a standard deviation for each condition around 0.2, and correlation between conditions around 0.7. Based on these numbers, an experiment with 20 participants will have a power of 0.9 for detecting a difference between the two conditions [20]. Any benefit of humming is likely a smaller effect than the detrimental impact of articulatory suppression. If we assume that the beneficial effect of humming is half the detrimental effect of articulatory suppression, then the mean proportion of correctly reported letters for humming trials would be 0.72. If we analyze the data with an ANOVA and two contrasts to test significance of the quiet and articulatory suppression trials (the classic finding) and the quiet and humming trials (the hypothesized effect), then a dependent ANOVA power analysis calculator [20] indicates that joint power of 0.9, across all three tests, can be reached with *n* = 72 participants. Given that there is some uncertainty about the size of a humming effect, we planned to gather data from *n* = 100 participants.

### 2.2 Participants

Participants were students at Purdue University who received course credit for their participation. Due to excess sign-ups, we ended up with data from *n* = 110 participants (69 females). Given the variables contributing to the power analyses above, such a study should have a power of around 0.98 to show both the detrimental effect of articulatory suppression and the hypothesized beneficial effect of humming. This analysis suggests that our experiment should have reasonable power to detect a potential beneficial impact of humming.

### 2.3 Method

The experiment was run online through a web browser using Javascript and HTML. After reading background and instructions, a participant started a trial by clicking on a *Next trial* button. A trial started with condition instructions, which were blocked for a sequence of 16 trials. The instructions for the Number, Quiet, and Humming conditions were, respectively, “Start saying 1 2 3 4 1 2 3 4…”, “Remain quiet”, and “Hum through your nose”. To avoid subvocalized phonological intrusions during humming trials, participants were instructed, “Do not hum a tune or mentally sing a corresponding tune.” The order of conditions was randomized for each participant. Two seconds after the condition instructions appeared, seven letters were presented for one second each. The letters were always: F, K, L, M, Q, R, and X, presented in a random order each trial. After the letter sequence, the participant stopped the Number or Humming task and was prompted to click on labeled buttons in the same order as the letters were presented on the trial. When ready, the participant started the next trial with a button press. No feedback was given on how well the participant performed on a given trial, but a summary of the performance for each condition was provided at the end of the experiment.

Data collection started on 02 February 2022 and ended on 12 February 2022.

### 2.4 Results

**Fig 1:**
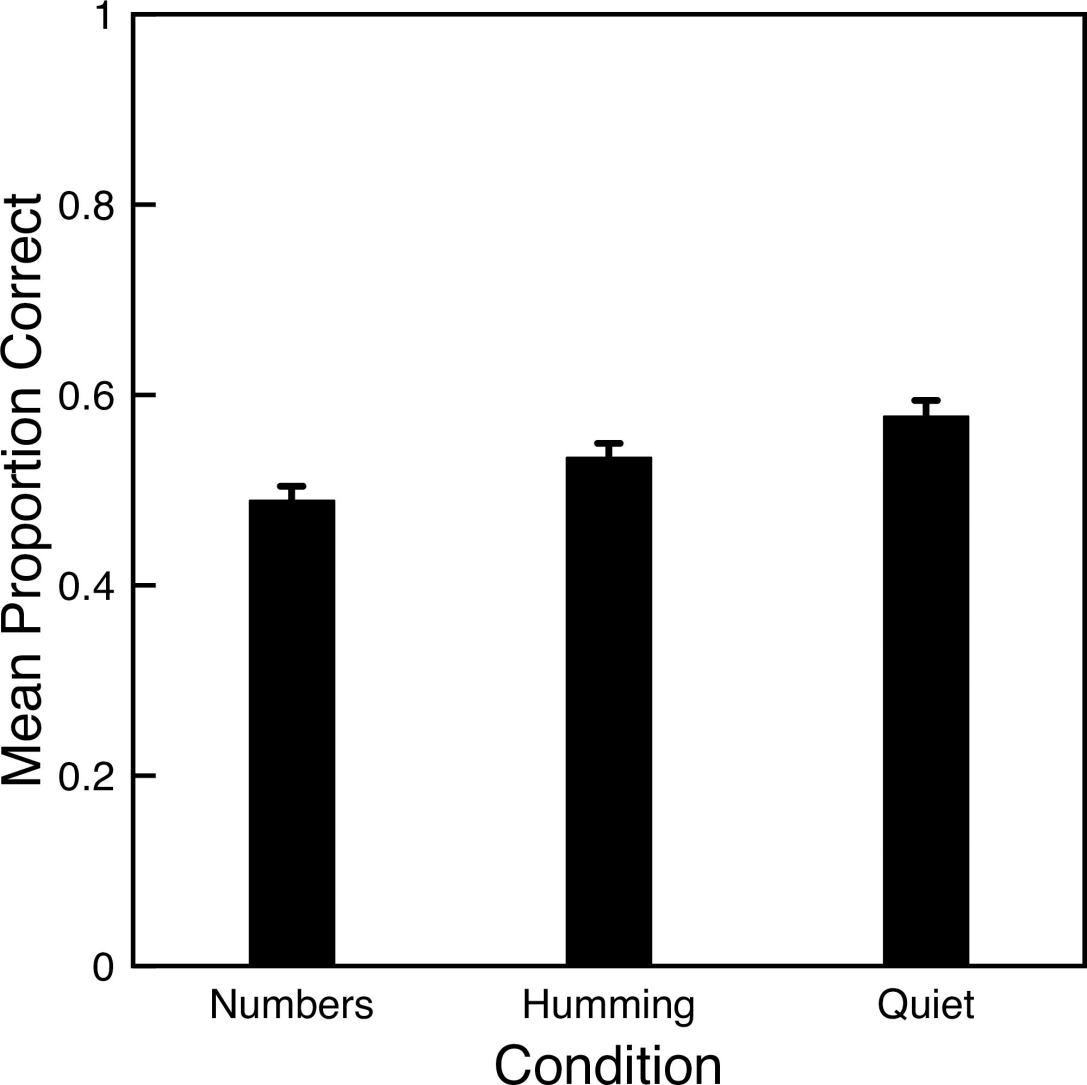
Mean proportion correct letter recall for Experiment 1. Error bars indicate one standard error of the mean for each condition.

Fig 1 shows the mean proportion of correct responses for each condition. A dependent ANOVA shows a significant difference across conditions (*F*(2, 218) = 14.62, *p*<0.001). Consistent with the classic result, more letters were correctly recalled for the quiet condition than for the articulatory suppression (Numbers) condition (*t*(218) = 5.41, *p*<0.001). Contrary to the hypothesized impact of humming, participants recalled fewer letters while humming than in the quiet condition (*t*(218) = 2.69, *p* = 0.008).

Overall, performance was lower than for previous studies (e.g., those used to guide the power analysis), and correlations between conditions were also lower (*r* = 0.36 for numbers and quiet, *r* = 0.42 for numbers and humming, and *r* = 0.62 for humming and quiet). Standard deviations were around 0.17, which is a bit lower than for previous studies.

### 2.5 Discussion

The deleterious effect of humming, compared to the quiet condition, was a surprise because theories of working memory [18] suggest that humming (which has no phonological components) would not interfere with items in the phonological loop. Without any impact of humming-induced NO, these theories would predict that performance during humming trials would be similar to performance during quiet trials. Nonetheless, the observed deleterious effect provides no support for the hypothesis that the enhanced levels of NO induced by humming improve short term memory performance.

## 3 Experiment 2

The detrimental impact of humming in Experiment 1 may be because humming utilizes resources of the phonological loop (despite theory suggesting otherwise), thereby interfering with letter recall similarly to the articulatory suppression condition. Such interference might overwhelm any benefits of humming and hide a cognitive benefit of humming-induced NO.

To reduce such possible interference, a second experiment used a basic measure of performance: speeded detection of a visual stimulus for the same conditions (Numbers, Humming, Quiet) as in Experiment 1. The multiple-resource theory of task interference [21] suggests that verbal/vocal tasks such as repeating numbers or humming should have very little interference with button responses to the onset of a visual stimulus. Thus, if the increased NO induced by humming improves cognitive performance, there should be faster response times for humming trials than for quiet trials.

### 3.1 Power analysis and participants

To identify an appropriate sample size, we noted that online studies of simple reaction (presumably under what corresponds to the quiet condition) have response times of around 300 milliseconds with standard deviations of around 50 ms [19]. Correlations between various conditions of a response time measure are often around *r* = 0.7 [22]. To generate a ballpark estimate of how much humming might speed up responses, we note that presentation of a valid cue before target onset can reduce response times by around 26 milliseconds [23]. To be cautious, we supposed that the benefit of humming might be half that size. With the dependent ANOVA power calculator at [20], we entered means of 300, 287, and 300 for the Numbers, Humming, and Quiet conditions, respectively with a standard deviation of 50 ms, and a correlation of 0.7. With these values, for the experiment to have a joint power of 0.9 for the combination of a significant ANOVA and a significant contrast comparing the Humming and Quiet conditions we need at least *n* = 102 participants. We recruited 104 participants (53 females) from the population of students at Purdue University.

### 3.2 Method

The experiment was run online through a web browser using Javascript and HTML. After reading background and instructions, a participant started a trial by pressing the *n* key on their computer keyboard. Similar to Experiment 1, instructions appeared for two seconds indicating whether the participant should say numbers out loud, hum, or remain quiet. These instructions were repeated for a block of 20 trials, with different block orders randomized for each participant. When the instructions disappeared, a fixation cross was shown for one second. After a variable delay (1 to 3 seconds) a green circle then appeared at the location of the fixation cross, and the participant pressed the *m* key on their keyboard as soon as they saw the circle. If the time between the onset of the circle and the participant’s response was too short (less than 100 milliseconds), a warning appeared that advised the participant to wait until the green circle appeared (to prevent anticipatory responses). Likewise, if the response time was too long (more than 750 milliseconds), a warning appeared to encourage the participant to respond faster. On other trials, the participant received a report of their response time on that trial. When ready, the participant started the next trial with a key press.

Data collection started on 28 March 2022 and ended on 24 April 2022.

### 3.3 Results

**Fig 2:**
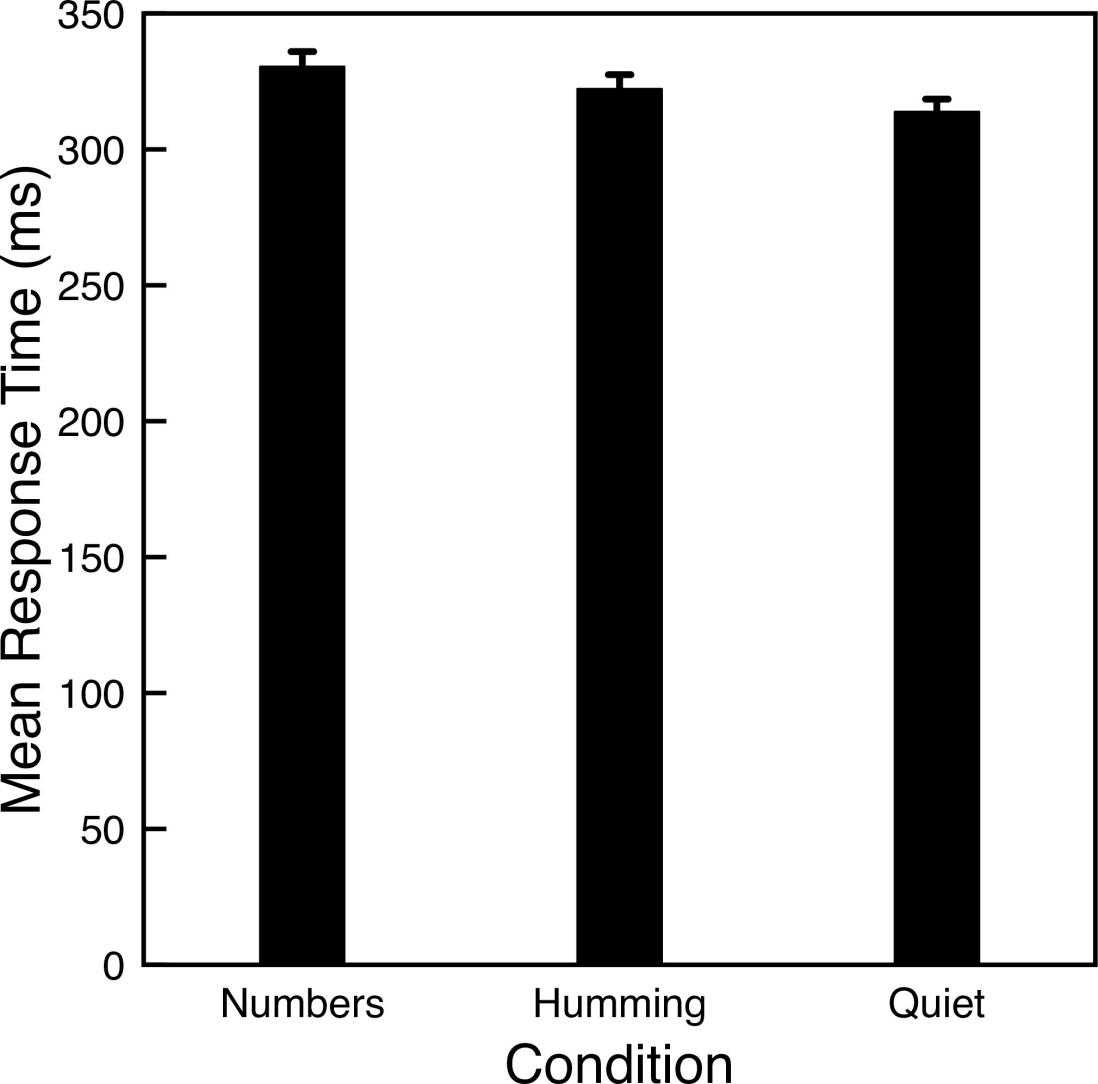
Mean response times for Experiment 2. Error bars indicate one standard error of the mean for each condition.

Fig 2 shows the mean response times for each condition. A dependent ANOVA shows a significant difference in response times across conditions (*F*(2, 206) = 14.34, *p*<0.001). Contrary to the hypothesized benefit of humming, participants were slower to detect the green circle while humming compared to the quiet condition (*t*(206) = 2.75, *p* = 0.006). Contrary to the prediction of the multiple-resource theory of task interference, saying numbers out loud also led to slower response times than the quiet condition (*t*(206) = 5.35, *p*<0.001).

### 3.4 Discussion

Similar to Experiment 1, the study of simple response time suggests that humming interferes with cognitive performance. We should note that our finding is not the first to contradict the multiple resources theory [24]. At any rate, if there is any benefit to humming on a simple detection task (e.g., by providing NO to the brain) it is overwhelmed by the (small) interference caused by humming.

## 4 Experiment 3

Next, we investigated whether humming might impact emotional processing. We had participants perform the same tasks (Numbers, Humming, Quiet) before evaluating their emotional reactions to a photo.

### 4.1 Power analysis and participants

To identify an appropriate sample size, we used the findings in [13] to identify an estimated effect size. They reported that an active meditation protocol, which included humming, increased positive affect and decreased negative affect. With the provided statistics, we derived a standardized effect size of *d* = 0.6 for positive affect (we used this effect size rather than the larger standardized effect for negative affect, because we want to be conservative about the magnitude of the effect). If such an effect were found for the Humming condition compared to the Quiet condition in our study, the joint power for a dependent ANOVA and a contrast between the Humming and Quiet conditions would have a power of 0.9 for a sample of *n* = 62. We ended up gathering data from 206 students at Purdue University. We removed the data for 6 participants who were too young to give consent and the data for 1 participant who reported misunderstanding the instructions. The resulting sample size of *n* = 199 (127 females) should have power close to 1 for detecting significant effects of the hypothesized magnitude.

### 4.2 Method

The experiment was run online through a web browser using Javascript and HTML. After reading background and instructions, a participant started a trial by pressing a *Start Next Trial* button. Similar to Experiments 1 and 2, each trial began with brief instructions appearing for two seconds that indicated whether the participant should say numbers out loud, hum, or remain quiet. These instructions were repeated for a block of 20 trials, and the blocks were presented in a random order that varied across participants. The instructions were replaced by a photo that was shown for five seconds. Participants were requested to continue the task during the presentation of the photo and to stop when the photo disappeared. At the offset of each photo, participants were prompted to use sliders to indicate the valence (negativity/positivity, on a scale running from -100 for Negative to +100 for Positive), arousal (overall strength of the emotional reaction, on a scale running from 0 for Low to +100 for High), and fear (on a scale running from 0 for None to +100 for Much) engendered by the photo.

Each block of 20 trials included 5 neutral photos, 5 positive photos, 5 negative photos, and 5 fearful photos randomly selected from the GAPED database [25], which validated that the photos have the intended interpretations. The photos were presented in a random order within each block and no photo was repeated across the experiment for a participant. Different participants were presented with different randomly selected photos from the database.

Data collection started on 28 September 2022 and ended on 03 October 2022.

### 4.3 Results

**Fig 3:**
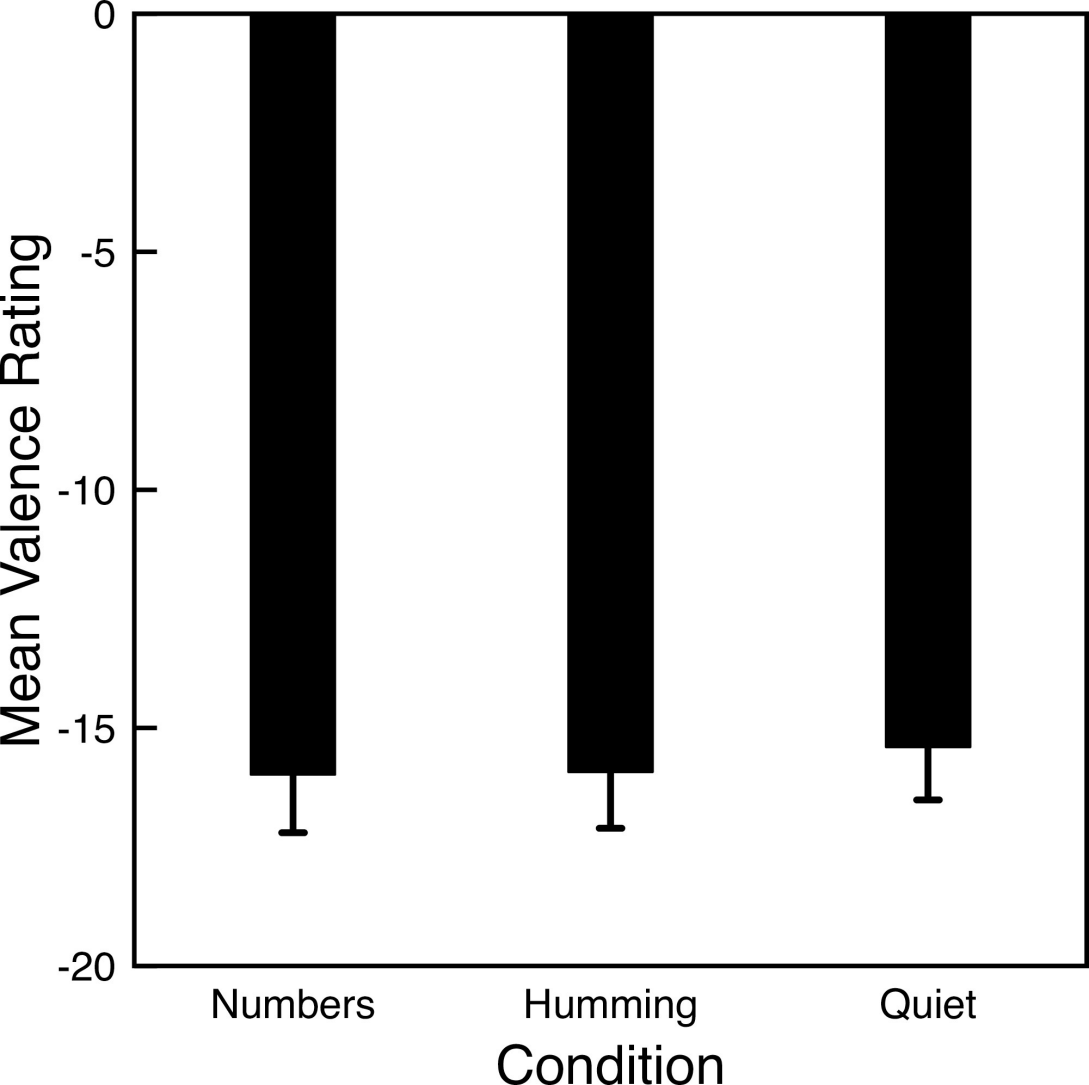
Mean valence ratings for Experiment 3. Lower bars indicate stronger negative ratings. Error bars indicate one standard error of the mean for each condition.

We first consider the valence scores. The ratings were on a scale from -100 (very negative) to +100 (very positive). For each participant we computed the average valence score across all photos for each condition (Fig 3). The mean valence scores are negative largely because half of the photos were intended to produce negative valence (negative and fearful photos) and only one quarter of the photos were intended to produce positive values. A dependent ANOVA shows no significant difference in mean valence ratings across task conditions (*F*(2, 396) = 0.184, *p* = .83). Contrary to the hypothesized increase in positivity when humming, our participants rated the pictures as slightly more negative when humming compared to the quiet condition.

**Fig 4:**
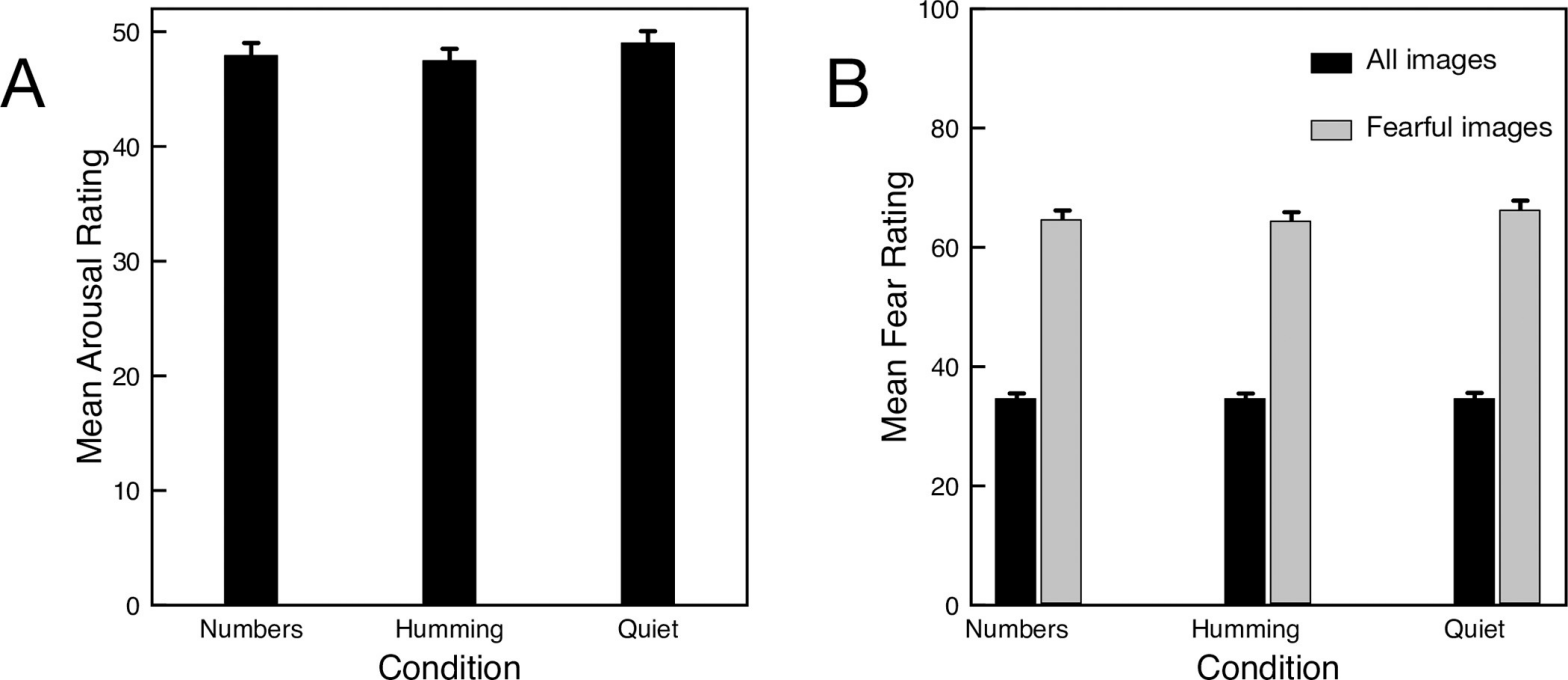
Mean arousal (A) and fear (B) ratings for Experiment 3. Higher values indicate more arousal/fear. Error bars indicate one standard error of the mean for each condition.

As exploratory analyses, we also considered possible impacts of humming on the arousal and fear ratings (Fig 4). Again, a dependent ANOVA shows no significant difference between the conditions. For the arousal ratings, *F*(2, 396) = 2.47, *p* = .09, with humming showing just a slightly lower mean arousal than the quiet condition. For the fear ratings, *F*(2, 396) = 0.014, *p* = .99, there is hardly any difference between conditions. We also looked specifically at the fear-inducing images, where there might be the largest effect of NO. Although the fearful images were rated higher on fear (Fig 4B) than other images, there was no significant difference between conditions, *F*(2, 396) = 1.62, *p* = 0.20.

### 4.4 Discussion

Overall, we find no significant impact of humming on emotional processing.

## 5 Experiment 4

For a final investigation, we returned to the speeded detection task used in Experiment 2. Contrary to our expectations, Experiment 2 found slower response times during humming than during the Quiet condition. It could be that the effort required to continuously hum prevented participants from fully attending to the stimulus presentation task, thereby leading to longer response times compared to the Quiet condition. Thus, it is possible that a benefit from release of paranasal NO was masked by a larger interference effect derived from humming.

In Experiment 4 we temporally separated the humming and detection tasks to allow participants to fully attend to the presentation of the visual stimulus. Previous work [26] measured the time course of nasal NO concentrations after humming during a single breath. NO measurements peaked at around one second of humming and then gradually decreased over the subsequent 10 seconds. They also found that NO concentration decreased over repeated humming breaths; however, NO concentrations during humming were still substantially higher than during silent breathing. Thus, we reasoned that any effect of paranasal NO on cognition should last several seconds after humming.

### 5.1 Participants

In addition to separating the visual detection task from the secondary task (Humming, Numbers, Quiet) in time, we were also concerned that the on-line nature of our experiments might have limited our ability to detect a positive effect of NO. In particular, some participants might have been congested when doing the experiment, which could impair release of paranasal NO. Moreover, we had no way to monitor whether participants actually followed the instructions and hummed when requested. To address these concerns, at the end of the experiment we asked participants about their congestion status (Very congested, Partially congested, Not congested) and whether they followed the instructions to hum (Always hummed, Usually hummed, Rarely hummed, and Never hummed) to be able to analyze only data from participants who complied with the instructions and were free from congestion.

### 5.2 Methods

Experiment 4 was similar to Experiment 2, with the key difference that each trial started with a request to perform the secondary task (Numbers, Humming, Quiet) for five seconds. After five seconds, the participant was asked to stop performing the task. One second later, a fixation cross appeared for one second. The fixation cross disappeared and after another 1–3 seconds the green circle appeared until the participant made their detection response.

Data collection started 09 February 2024 and ended 16 February 2024.

### 5.3 Results

**Fig 5:**
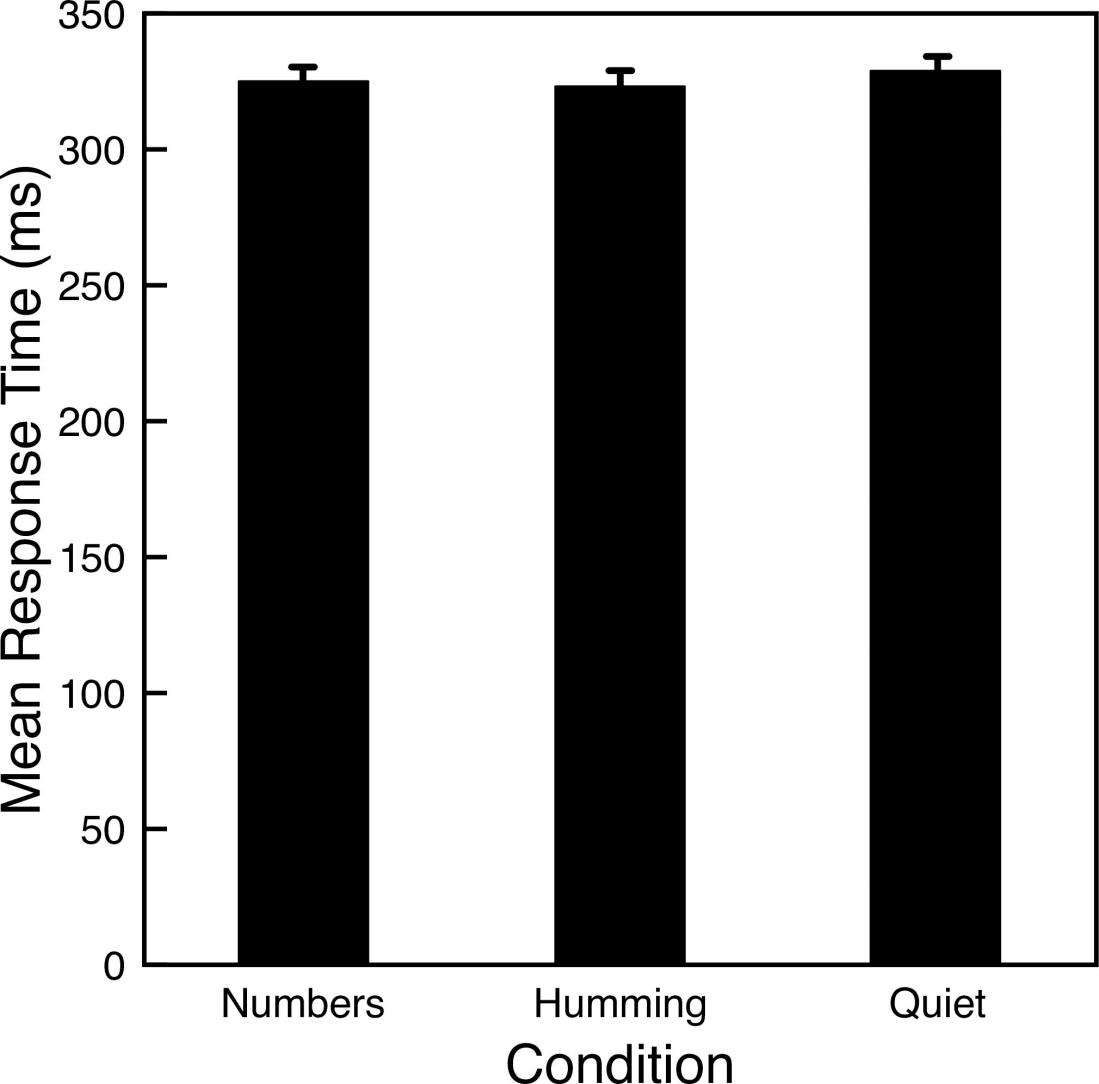
Mean response times for Experiment 4. Error bars indicate one standard error of the mean for each condition.

A total of 265 participants completed the experiment. From the full data set *n* = 91 participants reported both no congestion and that they always hummed when requested. Fig 5 shows that the mean response time for these participants hardly varied across conditions. Although the mean response time over the humming trials (323.5 ms) is slightly faster than the mean response time for quiet trials (328.1 ms), a one-way ANOVA finds no significant difference for any of the conditions (F(2, 180) = 0.747, p = 0.475). We repeated the analysis for the full 265 participants and, again, found no significant differences between conditions.

### 5.4 Discussion

Our modification of the speeded response detection task seems to have successfully removed the interfering effects of humming found in Experiment 2. However, we still find no significant impact of humming on response time. If there is any effect of humming on this cognitive task, it seems to be very small.

## 6 Conclusions

There is convincing evidence that humming increases nasal NO in humans and that NO has many positive benefits for cognition in non-human animals. Should such a benefit be found also in humans, it might provide a quick and easy way to enhance cognition for a variety of situations and might help explain previously reported benefits of humming as part of meditative practices. Contrary to the prediction, two of our experiments found that humming produced worse cognitive performance (poorer recall and slower response times) than a quiet condition. In a follow-up experiment, we separated the humming task from the stimulus presentation to remove any interference effects, but still found no indication of a positive effect on performance. Thus, if there are any cognitive benefits of nasal NO, they seem to be very small or apply to processes that we did not study. The negative interference of humming also contradicts popular theories of information processing [18,21]. Despite having no phonological component, humming interfered with short-term memory of verbal information; and despite requiring no visual resources, humming interfered with speeded detection of a spot of light. Regardless of the processing details, our studies suggest that humming is not an effective way to improve cognition, at least over the short term. Likewise, animal studies suggested that NO levels influence emotional processing [14,15]. Based on those studies, we expected that increased NO from humming would modulate emotional processing, especially by increasing positivity and reducing negativity. Our experiment finds no support for such an effect, despite using a much larger sample than previous studies.

One limitation of our experiments is that we did not measure nasal NO concentrations and we cannot estimate how much nasal NO might traverse to the brain. However, it is well established that humming increases nasal NO [9,10,27,28], so much so that NO levels during humming are often used as a screening test for some respiratory problems [29–31]. NO has the possibility to reach lower parts of the brain via the nose in a similar way as has been suggested for nasal spray drugs [32]. Nevertheless, it is not clear how much nasal NO might reach the brain and thereby influence cortical or subcortical processing. It could be that higher concentrations of NO (perhaps produced by particular types of humming) or longer periods of heightened NO concentrations would have positive benefits on cognition. Likewise, it could be that some people will have cognitive benefits from humming, but our studies suggest that such effects are small, on average.

On the other hand, there may be a deeper lesson here about potential manipulations of brain chemistry. Given the relative ease with which people can generate NO (e.g., by humming), if such activity had positive benefits, one can argue that such an advantage would already be utilized. One possibility is that a decrease of NO might impair system processing, but an increase of NO from normal levels would not enhance performance. Finally, if NO contributes to the many hypothesized roles in brain communication, it might have deleterious or facilitatory effects depending on the precise details of the stimuli, task, and test conditions, and therefore not have clear observable cognitive and emotional impacts. Likewise, the ubiquitous role of NO speaks against NO specifically influencing evaluations of fear-inducing images because it would suggest a very restricted impact of NO to the content of images and the evaluation process.

Regardless of these details, our experiments suggest that, at least over the short term, humming does not positively impact cognitive processing and seems to have no influence on emotional processing.
